# The Economic and Clinical Burden of Pediatric Obesity Within a Universal Health Coverage System in Thailand: A 9-Year Nationwide Analysis of 14.5 Million Hospitalizations

**DOI:** 10.3390/diseases14070242

**Published:** 2026-07-04

**Authors:** Tran Cong Ly, Suchaorn Saengnipanthkul, Phanthila Sitthikarnkha, Leelawadee Techasatian, Kaewjai Thepsuthammarat, Pope Kosalaraksa, Rattapon Uppala

**Affiliations:** 1 Doctor of Philosophy Program in Clinical Sciences, Faculty of Medicine, Khon Kaen University, Khon Kaen 40002, Thailand; ly.t@kkumail.com; 2 Department of Pediatrics, Faculty of Medicine, Can Tho University of Medicine and Pharmacy, Can Tho 94120, Vietnam; 3 Department of Pediatrics, Faculty of Medicine, Khon Kaen University, Khon Kaen 40002, Thailand; puntsi@kku.ac.th (P.S.); leelawadee@kku.ac.th (L.T.); popkos@kku.ac.th (P.K.); rattapon@kku.ac.th (R.U.); 4 Clinical Epidemiology Unit, Faculty of Medicine, Khon Kaen University, Khon Kaen 40002, Thailand; ckaewj@kku.ac.th

**Keywords:** hospitalization, health care utilization, in-hospital mortality, obesity, pediatrics

## Abstract

Background: While pediatric obesity prevalence is rising, the association between ICD-coded obesity, healthcare resource utilization, and inpatient outcomes in middle-income countries remains poorly quantified. This study examined inpatient diagnostic patterns, resource utilization, and in-hospital mortality among hospitalized pediatric patients with ICD-coded obesity in Thailand’s Universal Coverage scheme during a 9-year period. Methods: We analyzed nationwide inpatient administrative data from January 2015 to December 2023 for children aged 1 month to <18 years. ICD-coded obesity was defined using ICD-10-TM codes recorded as either a principal diagnosis or a comorbidity. Outcomes included length of stay, hospital costs, and in-hospital mortality. Univariable and multivariable regression models were used to estimate associations between ICD-coded obesity and inpatient outcomes, with adjustment for age, sex, region, hospital level, admission year, and disease categories. Results: Among 14,483,566 hospitalized children, 42,168 had ICD-coded obesity. Notably, 95.7% of children with ICD-coded obesity were recorded as a comorbidity rather than the primary reason for admission. Children with ICD-coded obesity as a comorbidity had 156.8% higher median hospital costs. Across all major categories of common acute diseases (respiratory, intestinal, digestive), children with ICD-coded obesity had significantly higher median costs and longer length of stay compared to children without ICD-coded obesity. In regression analyses, ICD-coded obesity remained associated with longer length of stay (adjusted ratio, 1.21; 95% CI, 1.16–1.26; *p* < 0.001) and higher hospitalization cost (adjusted cost ratio, 1.42; 95% CI, 1.32–1.53; *p* < 0.001). The association with in-hospital mortality was observed in the unadjusted model but was attenuated after adjustment and was not statistically significant (adjusted odds ratio, 1.14; 95% CI, 0.89–1.45; *p* = 0.303). Conclusions: In Thailand’s national universal coverage scheme, ICD-coded obesity was associated with greater inpatient resource utilization, especially longer length of stay and higher hospitalization costs. These findings support the need for weight-aware inpatient management and adjusted funding models for hospitals treating this higher-resource-utilization subgroup.

## 1. Introduction

Childhood obesity has evolved from a long-term public health concern into a condition increasingly associated with healthcare system strain. While global projections suggest a relentless rise in the burden of obesity in children and adolescents [1], the trajectory is particularly aggressive in rapidly transitioning middle-income countries [2]. Thailand reflects this pattern. Over the past two decades, obesity prevalence among school-age children has increased from about 6% to 15% [3]. This growing burden supports the need to understand not only long-term cardiometabolic outcomes but also the immediate impact of this epidemic on the acute care infrastructure, specifically within the context of Universal Health Coverage (UHC), which remains critically under-examined.

Pediatric obesity is now viewed as a chronic, complex disease associated with multisystem comorbidity and functional vulnerability, not simply as excess weight [4]. In addition to cardiometabolic consequences, obesity is linked to conditions commonly managed in hospitals, including asthma and other respiratory disorders, sleep-disordered breathing, and hepatometabolic disease [4]. Beyond chronic comorbidity, evidence from acute infectious conditions suggests that children with obesity may experience greater inpatient burden in selected clinical settings. For example, a systematic review and meta-analysis of pediatric influenza studies reported that children with obesity had higher odds of hospitalization, and that hospitalized children with obesity had higher odds of adverse inpatient outcomes [5]. However, evidence remains limited in middle-income countries, particularly within universal healthcare systems, where administrative data can provide insight into diagnosis patterns, resource utilization, and inpatient outcomes at the population level.

Beyond clinical outcomes, obesity introduces significant economic burden during hospitalization. Altered pharmacokinetics and dosing uncertainty have been reported across multiple drug classes in children with obesity. This can increase the risk of under-treatment or toxicity and may affect length of stay and resource use [6,7]. Several studies have also reported longer hospital stays and higher costs among pediatric inpatients with obesity compared with normal-weight peers. In high-income settings, administrative and hospital-based studies suggest that obesity, particularly when coded as a comorbidity, is associated with longer stays and higher costs or charges for common pediatric admissions [8]. Another hospital-based study reported that pediatric inpatients with obesity had about 15% longer length of stay and 19% higher total hospital costs than normal-weight patients with the same primary diagnoses, after adjustment for demographics and other factors [9]. Together, these findings suggest that obesity may modify inpatient utilization and outcomes across diverse pediatric conditions, not only admissions directly attributed to obesity.

However, important evidence gaps remain. First, much of the inpatient evidence comes from single-disease cohorts or from health systems in high-income countries, which may not generalize to middle-income settings with different epidemiology, care pathways, and financing [8]. Second, few studies have examined how obesity relates to in-hospital outcomes across broad diagnostic categories in routine practice. This information is needed to identify where incremental burden is greatest and where targeted inpatient screening, treatment optimization, and referral pathways may have the most value. Third, coding position may capture different clinical contexts. Obesity coded as a principal diagnosis likely reflects a different reason for admission than obesity coded as a comorbidity, yet this distinction has rarely been used to interpret population-level hospitalization burden [10].

To address these gaps, we analyzed nationwide hospitalization data from Thailand (2015–2023). We aimed to characterize the landscape of obesity-associated diagnoses, and estimate the association between ICD-coded obesity and length of stay, hospital costs, and in-hospital mortality across major pediatric disease groups. This study provides policy-relevant evidence on where inpatient burden associated with ICD-coded obesity is greatest in Thailand and informs hospital-based strategies that align with pediatric obesity as a chronic, multisystem condition requiring active clinical management.

## 2. Materials and Methods

### 2.1. Study Design and Data Source

We conducted a nationwide retrospective cohort study using the National Health Security Office (NHSO) administrative database, which covers Thailand’s UHC scheme, a system providing healthcare to approximately 72% of Thai population. The database captures all inpatient admissions with diagnoses coded according to the International Classification of Diseases, 10th Revision, Thai Modification (ICD-10-TM). The study period was 1 January 2015 to 31 December 2023.

### 2.2. Study Population and Index Admission

The study included children aged 1 month to younger than 18 years with at least one inpatient admission during the study period. To avoid over-representation of frequent admissions, we utilized a patient-level analysis and counted each child once. Children were identified using an encrypted patient identifier in the NHSO database, which allowed admissions for the same child to be linked across the study period and across hospitals.

Obesity was defined as any child with an ICD-10-TM code in the E66 code family recorded in any diagnosis field (principal or secondary) during the study period, including E66.0–E66.9 and any ICD-10-TM extension codes beginning with E66 when present in the database. Because anthropometric data were unavailable, this definition represents ICD-coded obesity. For these children, the index admission was defined as the first hospitalization in which E66.x was recorded. For children without any record of E66.x across the entire 9-year study period, the index admission was their first recorded hospitalization during the study period. Although admissions could be linked across hospitals using the encrypted unique patient identifier, transfer-related admissions were not collapsed into a single episode of care because the analytic unit was the patient-level index admission. Subsequent or recurrent admissions after the selected index admission were excluded from the main outcome analysis. We excluded records with incomplete key variables required for analysis.

### 2.3. Variables and Outcomes

We extracted age at admission, sex, geographic region, and hospital service level from the NHSO database. Hospital service level was classified as primary, secondary, tertiary, or private according to NHSO facility classification [11]. Admission year was also extracted to partially account for temporal changes in hospitalization patterns, healthcare costs, and service use across the study period. Age was categorized into four developmental groups: <1 year, 1–<5 years, 5–<13 years, and 13–<18 years. These categories were selected to reflect major pediatric developmental stages, including infancy, early childhood or preschool age, school-age and pre-adolescent childhood, and adolescence.

ICD-coded obesity status was categorized into three mutually exclusive groups for analysis: (1) obesity recorded as the principal diagnosis on the index admission (E66.x in the principal diagnosis field), (2) obesity recorded as a comorbidity on the index admission (E66.x recorded as a secondary diagnosis but not the principal diagnosis) and (3) non-obesity (no E66.x recorded on the index admission, and no E66.x recorded across the study period). Cases with obesity due to endocrine, genetic, or syndromic causes were not excluded if E66.x was recorded, because these etiologies could not be separately verified without chart-level or anthropometric data. They could only be identified if corresponding diagnosis codes were also recorded. BMI, BMI-for-age z-scores, and obesity class were not available in the administrative database.

Diagnoses used for descriptive ranking and comorbidity profiling were taken from the index admission. For comorbidity profiling, diagnoses were counted at the patient level so that each diagnosis was counted once per child, regardless of the number of admissions.

To assess associations between ICD-coded obesity and inpatient outcomes across major pediatric disease contexts, we grouped index admissions based on the principal ICD-10 chapter of major pediatric conditions. Disease groups were defined as: respiratory system infections (J00–J22, J40–J47, J85–J86), intestinal infections (A00–A09), other infections (A15–A74, B00–B99), digestive system diseases (K00–K93, R10–R19), arthropod-borne viral fevers (A75–A79, A90–A99), and neoplasms (C00–C97, D00–D48).

Hospital resource utilization outcomes were length of stay (days) and total hospital costs, presented in US Dollars (USD) and International Dollars (Purchasing Power Parity, PPP) (Table S1) based on World Bank conversion factors for 2023, which was the final year of the study. The cost field reflected the total charge or paid amount recorded for the admission. Costs were first recorded in Thai baht and converted to USD and PPP-adjusted international dollars using 2023 World Bank conversion factors. Costs were not additionally adjusted for inflation across admission years. Because the database contained recorded admission-level charges or paid amounts instead of standardized itemized economic costs, no additional inflation adjustment was applied. Admission year was included in the multivariable models to partially account for temporal changes in cost structure and healthcare utilization. In-hospital mortality was determined from the discharge status of the index admission.

### 2.4. Statistical Analysis

Analyses were performed using Stata version 18 (StataCorp LLC, College Station, TX, USA). Figures were produced in R version 4.5.1 (R Foundation for Statistical Computing, Vienna, Austria). Continuous variables were summarized as medians with interquartile ranges (IQRs). Categorical variables were summarized as counts and percentages.

We ranked diagnosis codes from the index admission in two ways. Among children whose index admission listed obesity as the principal diagnosis, we ranked secondary diagnoses to identify the 10 most frequent comorbidities. Among children whose index admission listed obesity as a secondary diagnosis, we ranked principal diagnoses to identify the 10 most frequent reasons for admission. We compared length of stay, costs, and in-hospital mortality across the three obesity status groups overall and within prespecified disease groups. We also stratified comparisons by age group.

For skewed continuous outcomes, we used the Kruskal–Wallis test for three-group comparisons and the Wilcoxon rank-sum test for two-group comparisons. For categorical outcomes, the chi-square test or Fisher exact test was used, as appropriate. To evaluate the association between ICD-coded obesity and inpatient outcomes, univariable and multivariable regression models were fitted separately for length of stay, hospitalization cost, and in-hospital mortality. Univariable models included ICD-coded obesity as the only independent variable. Multivariable models were adjusted for age, sex, region, hospital level, admission year, and disease categories. Disease categories were included as separate binary covariates because more than one disease category could be recorded for the same admission. Negative binomial regression was used for length of stay because this outcome was recorded as count data and showed right-skewed dispersion. A Gamma generalized linear model with a log link was used for hospitalization cost because cost data were positive and right-skewed. Logistic regression was used for in-hospital mortality. Robust standard errors clustered at the hospital level were used in the regression models. Effect estimates were reported as length-of-stay ratios for length of stay, cost ratios for hospitalization cost, and odds ratios for in-hospital mortality, with 95% confidence intervals. All tests were two-sided with a significance level of 0.05.

## 3. Results

### 3.1. National Cohort Profile and the Coding Position

Between 2015 and 2023, a total of 14,483,566 pediatric patient-level index admissions were recorded nationwide. In this cohort, 42,168 patients had ICD-coded obesity, with a mean age of 9.6 ± 4.5 years. The largest absolute number of obesity-coded index admissions was observed among children aged 5–<13 years; however, this category spanned a wider age interval than the other groups and should not be interpreted as an age-specific hospitalization rate. This cohort was predominantly male (63.6%) and concentrated in urbanized regions, with Bangkok and Central Thailand accounting for nearly half of all cases (46.4%). A defining characteristic of the cohort was the coding position. ICD-coded obesity was recorded as a comorbidity in 95.7% of cases, whereas it was the principal reason for admission in only 4.3%. Furthermore, 58.2% of these resource-intensive admissions occurred in tertiary-level hospitals, signaling a high level of clinical complexity, as summarized inTable 1.

### 3.2. The Diagnostic Landscape: From Metabolic Disease to Acute Infection

**Figure 1:**
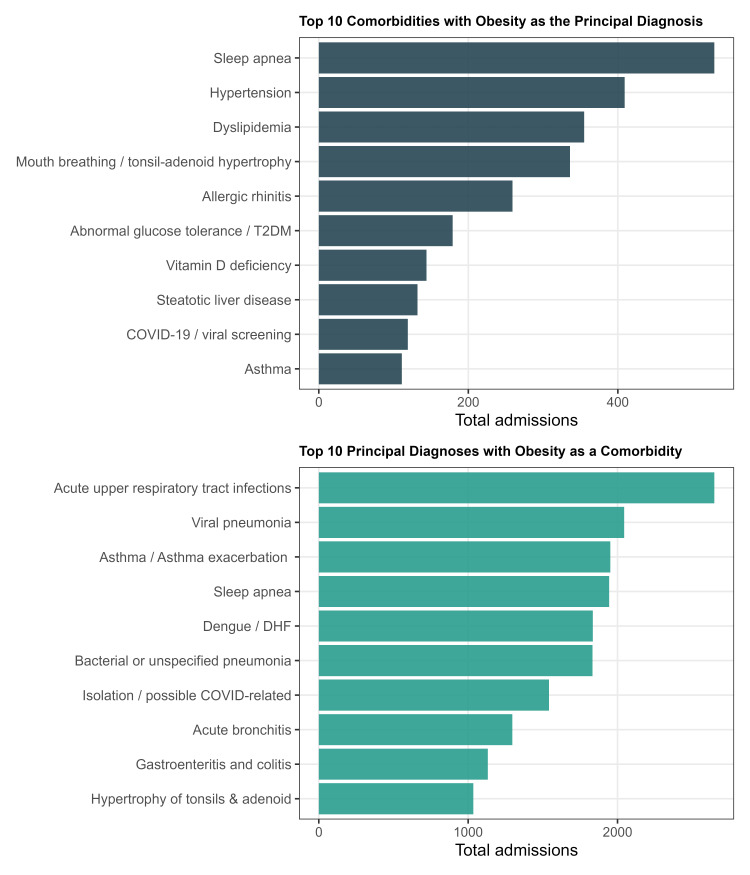
Top 10 Diagnoses in Admissions with ICD-Coded Obesity as The Primary Diagnosis and as A Comorbidity. Abbreviation: T2DM, Type 2 diabetes mellitus; DHF, Dengue Hemorrhagic Fever.

The study revealed two distinct epidemiological signatures for pediatric ICD-coded obesity, categorized by the coding position of the diagnosis (Figure 1). In cases where obesity was the principal diagnosis (*n* = 1827), the co-occurring disease profile was dominated by a high-density cluster of chronic metabolic and upper airway complications. Obstructive sleep apnea (OSA) was the most prevalent comorbidity, followed by hypertension. Significant metabolic complications, including dyslipidemia, type 2 diabetes mellitus, and steatotic liver disease, were predominantly ranked among the top 10 co-diagnoses.

In contrast, when obesity was recorded as a comorbidity (*n* = 40,341), admissions commonly involved acute infectious and respiratory illnesses. While OSA remained a common secondary finding, the principal reasons for hospital entry were dominated by acute upper respiratory tract infections. Pneumonia and asthma accounted for a substantial proportion of admissions, highlighting the synergy between excess adiposity and pulmonary vulnerability. Notably, dengue fever and dengue hemorrhagic fever were among the leading principal diagnoses, suggesting that in endemic middle-income settings like Thailand, obesity complicates the management of severe viral infections.

### 3.3. Hospital Utilization and In-Hospital Outcomes

**Figure 2:**
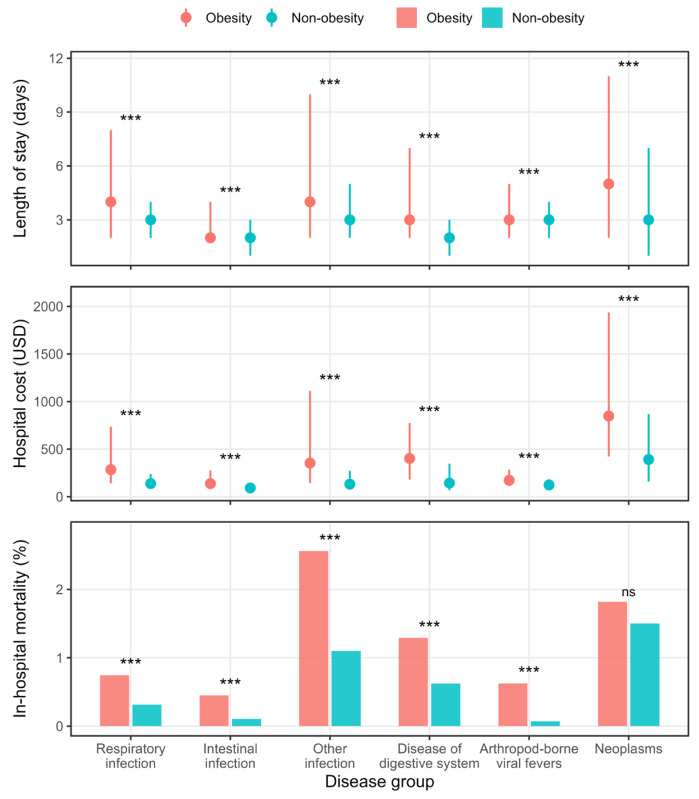
Hospital Utilization and Outcomes by ICD-Coded Obesity Status Across Common Hospitalized Pediatric Diseases. Abbreviation: ns, not significant. *** *p* < 0.001.

ICD-coded obesity was associated with greater hospital resource utilization in unadjusted comparisons, as shown in Figure 2 and Table 2. Obesity was associated with a significant increase in both median costs and length of stay (LOS). Children with obesity as the primary diagnosis had a slightly longer median LOS than the other groups (4 days, IQR 1–9). Hospitalization costs were highest when obesity was coded as a comorbidity, with a median expense of 273 USD (156.8% higher than non-obesity group). Neoplasms recorded the highest absolute costs (~848 vs. 391 USD, *p* < 0.001) and a 66% increase in LOS. For digestive diseases, costs were nearly three times higher in the obesity group (402 vs. 144 USD, *p* < 0.001).

In unadjusted comparisons, in-hospital mortality was higher among children with ICD-coded obesity recorded as a comorbidity than among children without ICD-coded obesity overall (0.5% vs. 0.3%, *p* < 0.001). Higher unadjusted mortality was also observed in several acute disease categories. For example, in the other infections category, mortality was 2.6% among children with ICD-coded obesity compared with 1.1% among children without ICD-coded obesity. In respiratory infections among children aged 5–<13 years, mortality was 1.0% among those with ICD-coded obesity and 0.2% among those without ICD-coded obesity (*p* < 0.001) (Table 3). While mortality was high in absolute terms among children with neoplasms, this was the only category where the presence of obesity did not significantly alter the survival outcomes (1.8% vs. 1.5%; *p* = 0.356), as shown in Figure 2.

In regression analyses, ICD-coded obesity remained associated with longer length of stay and higher hospitalization cost after adjustment for age, sex, region, hospital level, admission year, and disease categories (Table 4). The adjusted length-of-stay ratio was 1.21 (95% CI, 1.16–1.26; *p* < 0.001), and the adjusted cost ratio was 1.42 (95% CI, 1.32–1.53; *p* < 0.001). In contrast, the association between ICD-coded obesity and in-hospital mortality was attenuated after adjustment and was not statistically significant (adjusted odds ratio, 1.14; 95% CI, 0.89–1.45; *p* = 0.303).

Negative binomial regression was used for length of stay, Gamma generalized linear models with a log link were used for hospitalization cost, and logistic regression was used for in-hospital mortality. Multivariable models were adjusted for age, sex, region, hospital level, admission year, and disease categories. Effect estimates are shown as length-of-stay ratios for length of stay, cost ratios for hospitalization cost, and odds ratios for in-hospital mortality, with 95% confidence intervals. Robust standard errors clustered at the hospital level were used. Abbreviations: CI, confidence interval.

### 3.4. Age-Stratified Patterns of Hospital Utilization and In-Hospital Outcomes

Age-stratified analyses suggested that differences in utilization and mortality became more apparent after the first year of life. From age 1–5 years and most clearly in the 5–<13-year group, ICD-coded obesity was associated with longer stays, higher costs, and higher unadjusted mortality in several respiratory, intestinal, and viral fever categories (Table 3). Among adolescents (13–18 years), the mortality gap was most pronounced in intestinal and other infections, where the mortality rate for those with obesity was more than double that of their non-obesity counterparts.

## 4. Discussion

In this nationwide analysis of pediatric hospitalizations in Thailand, ICD-coded obesity was recorded predominantly as a clinical comorbidity rather than the principal reason for admission, with the coding position effectively capturing different clinical contexts. Admissions where ICD-coded obesity was the principal diagnosis clustered significantly with chronic respiratory and cardiometabolic comorbidities. In contrast, admissions where ICD-coded obesity was coded as a comorbidity were dominated by acute infectious and respiratory illnesses. Across major pediatric disease groups, children with ICD-coded obesity had higher unadjusted resource utilization than children without ICD-coded obesity. These patterns, which became most apparent beyond infancy, support the classification of obesity as a cross-cutting factor in inpatient risk assessment and acute care pathways. In multivariable regression models, ICD-coded obesity remained associated with longer length of stay and higher hospitalization costs, while the association with in-hospital mortality was attenuated and was not statistically significant after adjustment.

School-age children accounted for the majority of obesity-related hospitalizations in our cohort, while infants contributed relatively few. Population-based data in Thailand show a similar age distribution, with obesity prevalence rising sharply across middle childhood. For example, the Southeast Asian Nutrition Survey II (SEANUTS II) reported obesity rates among Thai children of 0.9% in infants, 3.9% in toddlers, and 16.0% in those aged 7 to 12.9 years [12]. This pattern is consistent with global epidemiologic evidence showing that obesity is less frequent in early childhood and increases during the school years [13]. Adolescents in our hospitalized population also represented a substantial burden, though lower than that of school-age children, aligning with a prior study [14]. One possible explanation is that adolescents are hospitalized less often than younger children. Furthermore, because obesity is often under-documented in administrative discharge data, adolescents admitted for acute conditions may not consistently receive an obesity ICD code, potentially leading to an underestimation of the true inpatient burden in this demographic.

Diagnosis patterns differed markedly by coding position. When obesity was the principal diagnosis, frequent coexisting conditions clustered in cardiometabolic and chronic respiratory domains, including dyslipidemia, steatotic liver disease, type 2 diabetes, hypertension, OSA, and asthma. This pattern is consistent with admissions where obesity is the clinical focus and where the evaluation of metabolic complications may be more exhaustive. Steatotic liver disease in children is strongly linked to excess adiposity, and current evidence syntheses report a substantial burden in pediatric populations, especially among children with obesity [15]. Hypertension was frequent, aligning with the higher prevalence of hypertension among children with overweight or obesity. This supports blood pressure assessment as a high-yield inpatient screening target with significant relevance to longer-term cardiovascular risk [16,17]. Vitamin D deficiency was also common among obesity-principal admissions. Prior studies have reported lower 25(OH)D levels in children with obesity, though whether this represents a causal pathway or a marker of lifestyle and adiposity-related physiology remains a subject of debate [18,19].

When obesity was coded as a comorbidity, principal diagnoses reflected a broader spectrum of acute illness, particularly respiratory and infectious conditions such as pneumonia, acute bronchitis, viral infections, and gastroenteritis. Notably, dengue and dengue hemorrhagic fever were frequent principal diagnoses, a finding particularly relevant to middle-income tropical settings. Two interpretations are plausible. First, obesity may increase the severity of common infections through altered respiratory mechanics and impaired immune or inflammatory responses. Meta-analytic data in pediatric influenza support a worse prognosis among children with obesity, including severe outcomes among those hospitalized [5]. Additionally, children with obesity may face a higher risk of clinically important dehydration, which could exacerbate illnesses such as acute gastroenteritis [20]. Second, administrative datasets often under-document obesity, and it may be more likely to be captured during complex admissions for acute illness than in routine care, potentially accentuating the “obesity as comorbidity” phenotype in hospital data [9,21,22].

Across both coding contexts, obstructive sleep apnea ranked as the most common diagnosis, alongside upper airway conditions such as adenotonsillar hypertrophy. This pattern is consistent with sleep-disordered breathing as a central comorbidity in hospitalized children with obesity. Recent syntheses identify a high burden of OSA among children with excess adiposity, with adenotonsillar hypertrophy as a common correlate [23]. These findings should be interpreted as descriptive patterns in administrative coding rather than causal effects, and they may reflect differences in diagnostic evaluation and coding practices between obesity-focused admissions and admissions for acute illness [22,24].

Hospitalizations involving ICD-coded obesity were associated with greater inpatient resource utilization. The most robust signal was the incremental cost, which remained higher after adjustment for available covariates. Across major diagnostic categories, children with ICD-coded obesity had longer stays and higher costs than children without ICD-coded obesity in unadjusted comparisons, with particularly large cost differentials in neoplasms, digestive diseases, and other infections. Prior administrative analyses have similarly reported higher charges and longer stays among children with obesity hospitalized for infections, including higher rates of organ dysfunction [25]. These results align with broader evidence that childhood obesity is associated with higher healthcare spending [26,27].

The coding position added clinical context. When ICD-coded obesity was recorded as a comorbidity, costs and unadjusted mortality were at their highest. This pattern suggests that ICD-coded obesity is more likely to be documented during clinically complex admissions and may identify admissions with greater management complexity and disease severity. Pediatric critical care syntheses have reported higher mortality and longer hospital stay among critically ill children with obesity [28]. A multicenter cohort study also found that obesity was independently associated with higher PICU mortality after adjustment for other factors [29]. Several biological mechanisms likely contribute, including altered respiratory mechanics and a higher risk of respiratory decompensation. In large US administrative analyses, obesity has been independently associated with mechanical ventilation, bacteremia or septicemia, higher costs, and longer length of stay [30].

The higher mortality observed for infectious categories is biologically plausible. Childhood obesity is associated with immune dysregulation and chronic low-grade inflammation, which may impair host defense during acute infection [31,32]. A systematic review and meta-analysis in pediatric influenza has reported a worse prognosis for children with obesity, including an increased likelihood of ICU-level outcomes or death [5]. Medication management may further contribute. Pharmacokinetics can differ significantly in children with obesity, and dosing evidence remains limited for many drugs. This raises the possibility of suboptimal therapeutic exposure or toxicity risk, both of which could affect complications and length of stay [6,33].

Age-stratified analyses showed that utilization and mortality differences emerged mainly after infancy and were strongest in children aged 5 to 13 years. This pattern is expected, as diagnosing obesity in children younger than 2 years is challenging and less standardized [34]. In this age group, excess weight is more often attributable to secondary causes, such as genetic syndromes, monogenic obesity, or endocrine disorders [35], rather than lifestyle-related adiposity, and therefore may follow different clinical trajectories. Cardiometabolic and inflammatory effects accumulate with longer duration of adiposity, and obesity is more consistently recognized and coded after the first year of life [36].

The absence of a mortality difference for neoplasms, despite substantially higher costs, suggests that short-term inpatient mortality in pediatric oncology may be driven primarily by disease biology and treatment intensity. In this setting, obesity may contribute more to supportive care needs, procedures, and intensive monitoring than to immediate in-hospital mortality risk. Prior studies have reported mixed associations between obesity and mortality in pediatric cancer populations. A large cohort study reported worse long-term outcomes in some cancers among children with obesity at diagnosis [37]. In contrast, a systematic review in pediatric cancer and hematopoietic stem cell transplantation reported inconsistent findings across studies, with some showing higher mortality and others showing no difference [27]. Our outcome captured in-hospital mortality and did not assess long-term survival, relapse, or treatment toxicity, which may explain part of this discrepancy.

This study utilized a large nationwide administrative dataset and applied a patient-level index admission approach to characterize the inpatient burden of ICD-coded obesity. Several limitations should be considered. First, obesity was identified using ICD codes rather than measured anthropometrics, making under-coding and misclassification likely. The low prevalence of ICD-coded obesity suggests that the coded cohort may represent only a selected subset of children with obesity, particularly those whose obesity was clinically recognized, documented, or coded during more severe or complex admissions. Surveillance or documentation bias is also possible because children with more severe or complex admissions may have undergone more detailed clinical evaluation, increasing the likelihood that obesity and related comorbidities were recorded. BMI, BMI-for-age z-scores, and obesity class were unavailable; therefore, this study could not assess obesity severity or dose–response associations. Consequently, the findings should be interpreted as associations among hospitalized children with recorded obesity diagnosis codes, rather than estimates for all hospitalized children with measured obesity. Second, coding position can reflect documentation practices and billing priorities as much as clinical context, and no anthropometric validation of obesity coding was available in this dataset. Third, although multivariable models adjusted for available demographic, hospital, temporal, and disease-category covariates, residual confounding remains possible because the database lacked clinical severity measures, laboratory data, medication details, and detailed comorbidity severity. Fourth, costs were recorded in Thai baht and converted to USD and PPP-adjusted international dollars using 2023 World Bank conversion factors; however, they were not additionally adjusted for inflation across admission years. Costs reflect recorded charges, which may vary by facility, reimbursement practice, calendar year, and service structure. Admission year was included in adjusted models to partly account for temporal changes, but COVID-19-related disruptions to hospital utilization and cost patterns may still have influenced estimates. Fifth, the index-admission approach reduced overrepresentation of children with repeated admissions, but it may underestimate cumulative healthcare burden because recurrent admissions and post-discharge events were not captured. Although admissions could be linked across hospitals using an encrypted patient identifier, transfer-related hospitalizations were not collapsed into a single episode of care. Therefore, length of stay and cost estimates reflect the selected index admission and may not capture all care delivered across multiple facilities during the same illness episode. Sixth, age groups were selected to reflect developmental pediatric stages and were not equal-width intervals. Therefore, comparisons based on absolute counts may be influenced by the wider duration of the 5–<13-year category and should not be interpreted as age-specific hospitalization rates. Finally, the analysis was limited to the UHC scheme, and findings may not generalize to children covered by other insurance systems.

Our data position pediatric obesity, especially when recorded as a comorbidity, may serve as an inpatient risk flag. Hospitals may reduce preventable harm and costs by pairing acute care with structured identification of obesity, screening for high-yield comorbidities such as OSA and hypertension, and implementing weight-aware medication dosing workflows. The concentration of excess burden in school-age children supports the development of targeted inpatient pathways in this age group, including clear referral plans for outpatient follow-up and long-term management. Given the prominence of respiratory and infectious admissions, pragmatic strategies should include strengthening discharge planning for respiratory risk and ensuring appropriate vaccination and prevention counseling to improve continuity of care after hospitalization.

## 5. Conclusions

Using nationwide pediatric inpatient data from Thailand, we found that ICD-coded obesity was most often documented as a comorbidity and was associated with longer length of stay and higher hospitalization costs after adjustment for available covariates. In-hospital mortality was higher in unadjusted analyses, but this association was attenuated and was not statistically significant after multivariable adjustment. Diagnosis patterns differed by coding position, suggesting distinct clinical contexts when ICD-coded obesity was recorded as the principal diagnosis versus a comorbidity. These findings indicate that ICD-coded obesity identifies a subgroup of hospitalized children with greater inpatient resource utilization across a broad range of pediatric conditions, although causal inference is limited by the observational design and administrative nature of the data. These results support strengthening hospital-based identification, risk assessment, and referral pathways for children with obesity, alongside coordinated outpatient management after discharge. Ultimately, addressing this underrecognized administrative and clinical signal may help support the long-term fiscal and clinical sustainability of the UHC scheme in an era of rising pediatric obesity.

## Figures and Tables

**Table 1:** General Characteristics of Hospitalized Children with ICD-Coded Obesity.

Characteristics	Value
Age at diagnosis (years)	
Mean ± SD	9.6 ± 4.5
<1 year, *n* (%)	372 (0.9)
1–<5 years, *n* (%)	6880 (16.3)
5–<13 years, *n* (%)	22,036 (52.3)
13–<18 years, *n* (%)	12,880 (30.5)
Sex, *n* (%)	
Male	26,811 (63.6)
Female	15,357 (36.4)
Region, *n* (%)	
Bangkok	8093 (19.2)
Central	11,454 (27.2)
Northeast	11,220 (26.6)
North	5749 (13.6)
South	5652 (13.4)
Hospital level, *n* (%)	
Primary	2138 (5.1)
Secondary	15,062 (35.7)
Tertiary	24,545 (58.2)
Private	423 (1.0)
Obesity diagnosis, *n* (%)	
As a principal diagnosis	1827 (4.3)
As a comorbidity	40,341 (95.7)

Abbreviation: SD, standard deviation.

**Table 2:** Hospital Utilization and Outcomes by ICD-Coded Obesity Status.

	Obesity		Non-Obesity	*p*
	As Primary Diagnosis	As Comorbidity		
Length of hospital stay (days), median (IQR)	4 (1–9)	3 (2–6)	3 (2–4)	<0.001
Hospital cost (USD), median (IQR)	198.3 (117.8–445.4)	273.0 (129.3–603.4)	106.3 (60.3–212.6)	<0.001
In-hospital mortality, *n* (%)	1 (0.1)	197 (0.5)	39,417 (0.3)	<0.001

Abbreviation: IQR, interquartile range.

**Table 3:** Hospital Utilization and Outcomes by ICD-Coded Obesity Status Across Age Groups in Common Hospitalized Pediatric Diseases.

	<1 Year			1–<5 Years			5–<13			13–<18		
	Obesity	Non-Obesity	*p*	Obesity	Non-Obesity	*p*	Obesity	Non-Obesity	*p*	Obesity	Non-Obesity	*p*
Length of stay (days), Median (IQR)												
Respiratory infection	4 (3–7)	3 (2–5)	<0.001	3 (2–5)	3 (2–4)	<0.001	4 (2–8)	3 (2–4)	<0.001	8 (4–11)	4 (2–9)	<0.001
Intestinal infection	4 (2–6)	3 (2–4)	<0.001	3 (2–4)	2 (2–3)	<0.001	2 (2–4)	2 (1–3)	<0.001	3 (2–5)	2 (1–3)	<0.001
Other infection	4 (2–14)	3 (2–8)	0.254	3 (2–6)	3 (2–4)	<0.001	5 (2–10)	3 (2–5)	<0.001	6 (3–13)	3 (2–6)	<0.001
Disease of digestive system	4 (3–10)	3 (2–7)	0.072	3 (2–6)	2 (1–3)	<0.001	3 (2–7)	2 (1–3)	<0.001	3 (2–7)	2 (1–3)	<0.001
Arthropod-borne viral fevers	3 (2–3.5)	3 (2–5)	0.338	3 (2–5)	3 (2–4)	0.042	3 (2–5)	3 (2–4)	<0.001	3 (2–5)	3 (2–4)	<0.001
Neoplasms	3 (2–35)	3 (2–8)	0.698	3 (1–10.5)	3 (1–7)	0.047	5 (2–11)	3 (1–6)	<0.001	5 (2–11)	4 (1–7)	<0.001
Hospital cost (USD), Median (IQR)												
Respiratory infection	198.3 (117.8–428.1)	155.2 (94.8–275.8)	<0.001	195.4 (117.8–370.7)	129.3 (86.2–209.8)	<0.001	270.1 (137.9–727.0)	132.2 (83.3–252.9)	<0.001	637.9 (296.0–1321.8)	221.3 (103.4–632.1)	<0.001
Intestinal infection	181.0 (109.2–350.6)	112.1 (71.8–181.0)	<0.001	135.0 (86.2–244.2)	91.9 (63.2–143.7)	<0.001	132.2 (77.6–273.0)	80.5 (54.6–126.4)	<0.001	158.0 (86.2–379.3)	80.5 (54.6–137.9)	<0.001
Other infection	227.0 (120.7–1680.9)	163.8 (89.1–379.3)	0.041	178.1 (103.4–399.4)	114.9 (74.7–192.5)	<0.001	390.8 (152.3–1163.7)	129.3 (80.5–267.2)	<0.001	591.9 (235.6–1706.8)	195.4 (106.3–482.7)	<0.001
Disease of digestive system	281.6 (143.7–1445.3)	178.1 (77.6–609.2)	0.065	290.2 (120.7–675.2)	103.4 (60.3–227.0)	<0.001	393.7 (172.4–747.1)	137.9 (66.1–344.8)	<0.001	445.4 (244.2–859.1)	267.2 (89.1–399.4)	<0.001
Arthropod-borne viral fevers	204.0 (158.0–261.5)	146.5 (94.8–241.4)	0.312	175.3 (100.6–350.6)	123.6 (83.3–189.6)	<0.001	166.7 (109.2–287.3)	120.7 (83.3–175.3)	<0.001	175.3 (114.9–270.1)	126.4 (89.1–186.8)	<0.001
Neoplasms	198.3 (54.6–3310.1)	278.7 (103.4–850.5)	0.756	594.8 (321.8–1520.0)	362.0 (158.0–747.1)	<0.001	841.9 (416.6–1902.2)	367.8 (140.8–818.9)	<0.001	890.7 (479.9–2088.9)	511.5 (232.7–1155.1)	<0.001
In-hospital mortality, n (%)												
Respiratory infection	1 (0.5)	4223 (0.8)	0.593	19 (0.5)	2015 (0.1)	<0.001	52 (1.0)	1726 (0.2)	<0.001	19 (0.7)	2015 (1.0)	0.071
Intestinal infection	0 (0.0)	558 (0.2)	0.695	4 (0.5)	409 (0.1)	<0.001	2 (0.1)	384 (0.1)	0.547	6 (1.7)	305 (0.2)	<0.001
Other infection	1 (2.0)	2430 (1.8)	0.906	4 (0.7)	1303 (0.5)	0.483	32 (2.6)	1455 (0.9)	<0.001	27 (4.1)	1832 (2.0)	<0.001
Disease of digestive system	1 (4.0)	2015 (2.6)	0.670	10 (1.6)	1272 (0.5)	<0.001	47 (1.5)	1587 (0.4)	<0.001	13 (0.8)	1411 (0.6)	0.205
Arthropod-borne viral fevers	0 (0.0)	39 (0.4)	0.857	0 (0.0)	50 (0.1)	0.777	19 (1.0)	177 (0.1)	<0.001	0 (0.0)	107 (0.1)	0.458
Neoplasms	0 (0.0)	270 (2.1)	0.696	1 (1.0)	637 (1.1)	0.965	12 (1.6)	1102 (1.2)	0.428	11 (2.4)	1102 (2.3)	0.887

Abbreviation: IQR, interquartile range.

**Table 4:** Univariable and Multivariable Associations between ICD-Coded Obesity and Inpatient Outcomes.

Outcome	Univariable Analysis		Multivariable Analysis	
	Unadjusted Ratio (95% CI)	*p*-Value	Adjusted Ratio (95% CI)	*p*-Value
Length of stay	1.35 (1.30, 1.39)	<0.001	1.21 (1.16, 1.26)	<0.001
Hospital cost	2.33 (2.15, 2.53)	<0.001	1.42 (1.32, 1.53)	<0.001
In-hospital mortality	1.72 (1.33, 2.24)	0.003	1.14 (0.89, 1.45)	0.303

## Data Availability

Data cannot be shared publicly because of legal and ethical restrictions protecting sensitive patient-level information and the data use agreement established with the National Health Security Office (NHSO) of Thailand. Data are available from the corresponding author (SS) upon reasonable request and with the formal approval of the Human Research Ethics Committee of Khon Kaen University.

## Supplementary Materials

The following supporting information can be downloaded at: https://www.mdpi.com/article/10.3390/diseases14070242/s1, Table S1: Hospitalization Costs Across Age Groups, Disease Categories, and ICD-coded Obesity Status in USD and International Dollars (Int$).

## Abbreviations

The following abbreviations are used in this manuscript:

UHC	Universal Health Coverage
NHSO	National Health Security Office
ICD-10-TM	International Classification of Diseases, 10th Revision, Thai Modification
USD	United States Dollar
