# Supplementary material for: The Economic and Clinical Burden of Pediatric Obesity Within a Universal Health Coverage System in Thailand: A 9-Year Nationwide Analysis of 14.5 Million Hospitalizations
Source: Diseases. 2026 Jul 4;14(7):242. doi: 10.3390/diseases14070242 (PMC13408753; doi:10.3390/diseases14070242)
Supplement: Supplementary file 1 [file diseases-14-00242-s001.zip › diseases-4381072-supplementary.pdf]

Supplementary Materials

Table S1. Hospitalization Costs Across Age Groups, Disease Categories, and ICD-coded Obesity Status in USD and International Dollars (Int$).

Currency	Diseases	Obesity group	Overall	<1 year	1-<5 years	5-<13 years	13-<18 years
Int$ (PPP)	Respiratory infection	Non-obesity	450.8 (281.8, 779.6)	507.2 (310.0, 901.7)	422.7 (281.8, 685.6)	432.1 (272.4, 826.5)	723.2 (338.1, 2,066.3)
		Obesity	929.9 (460.2, 2,404.5)	648.1 (385.1, 1,399.5)	638.7 (385.1, 1,211.6)	882.9 (450.8, 2,376.3)	2,085.1 (967.4, 4,320.5)
	Intestinal infection	Non-obesity	300.6 (197.2, 479.0)	366.3 (234.8, 591.7)	300.6 (206.6, 469.6)	263.0 (178.5, 413.3)	263.0 (178.5, 450.8)
		Obesity	450.8 (272.4, 901.7)	591.7 (356.9, 1,145.9)	441.4 (281.8, 798.4)	432.1 (253.6, 892.3)	516.6 (281.8, 1,239.8)
	Other infection	Non-obesity	432.1 (263.0, 892.3)	535.4 (291.2, 1,239.8)	375.7 (244.2, 629.3)	422.7 (263.0, 873.5)	638.7 (347.5, 1,577.9)
		Obesity	1,155.3 (469.6, 3,634.9)	742.0 (394.5, 5,494.6)	582.3 (338.1, 1,305.6)	1,277.4 (497.8, 3,803.9)	1,934.8 (770.2, 5,579.1)
	Disease of digestive system	Non-obesity	469.6 (225.4, 1,136.5)	582.3 (253.6, 1,991.2)	338.1 (197.2, 742.0)	450.8 (216.0, 1,127.1)	873.5 (291.2, 1,305.6)
		Obesity	1,314.9 (591.7, 2,536.0)	920.5 (469.6, 4,724.4)	948.6 (394.5, 2,207.2)	1,286.8 (563.5, 2,442.0)	1,455.8 (798.4, 2,808.3)
	Arthropod-borne viral fevers	Non-obesity	403.9 (272.4, 601.1)	479.0 (310.0, 789.0)	403.9 (272.4, 619.9)	394.5 (272.4, 572.9)	413.3 (291.2, 610.5)
		Obesity	563.5 (366.3, 929.9)	666.9 (516.6, 854.7)	572.9 (328.7, 1,145.9)	544.8 (356.9, 939.2)	572.9 (375.7, 882.9)
	Neoplasms	Non-obesity	1,277.4 (516.6, 2,836.5)	911.1 (338.1, 2,780.2)	1,183.4 (516.6, 2,442.0)	1,202.2 (460.2, 2,676.9)	1,671.9 (760.8, 3,775.8)
		Obesity	2,770.8 (1,380.7, 6,330.5)	648.1 (178.5, 10,820.1)	1,944.2 (1,052.0, 4,968.6)	2,752.0 (1,361.9, 6,217.8)	2,911.7 (1,568.5, 6,828.3)

Currency	Diseases	Obesity group	Overall	<1 year	1-<5 years	5-<13 years	13-<18 years
USD	Respiratory infection	Non-obesity	137.9 (86.2, 238.5)	155.2 (94.8, 275.8)	129.3 (86.2, 209.8)	132.2 (83.3, 252.9)	221.3 (103.4, 632.1)
		Obesity	284.5 (140.8, 735.6)	198.3 (117.8, 428.1)	195.4 (117.8, 370.7)	270.1 (137.9, 727.0)	637.9 (296.0, 1,321.8)
	Intestinal infection	Non-obesity	91.9 (60.3, 146.5)	112.1 (71.8, 181.0)	91.9 (63.2, 143.7)	80.5 (54.6, 126.4)	80.5 (54.6, 137.9)
		Obesity	137.9 (83.3, 275.8)	181.0 (109.2, 350.6)	135.0 (86.2, 244.2)	132.2 (77.6, 273.0)	158.0 (86.2, 379.3)
	Other infection	Non-obesity	132.2 (80.5, 273.0)	163.8 (89.1, 379.3)	114.9 (74.7, 192.5)	129.3 (80.5, 267.2)	195.4 (106.3, 482.7)
		Obesity	353.4 (143.7, 1,112.0)	227.0 (120.7, 1,680.9)	178.1 (103.4, 399.4)	390.8 (152.3, 1,163.7)	591.9 (235.6, 1,706.8)
	Disease of digestive system	Non-obesity	143.7 (69.0, 347.7)	178.1 (77.6, 609.2)	103.4 (60.3, 227.0)	137.9 (66.1, 344.8)	267.2 (89.1, 399.4)
		Obesity	402.3 (181.0, 775.8)	281.6 (143.7, 1,445.3)	290.2 (120.7, 675.2)	393.7 (172.4, 747.1)	445.4 (244.2, 859.1)
	Arthropod-borne viral fevers	Non-obesity	123.6 (83.3, 183.9)	146.5 (94.8, 241.4)	123.6 (83.3, 189.6)	120.7 (83.3, 175.3)	126.4 (89.1, 186.8)
		Obesity	172.4 (112.1, 284.5)	204.0 (158.0, 261.5)	175.3 (100.6, 350.6)	166.7 (109.2, 287.3)	175.3 (114.9, 270.1)
	Neoplasms	Non-obesity	390.8 (158.0, 867.8)	278.7 (103.4, 850.5)	362.0 (158.0, 747.1)	367.8 (140.8, 818.9)	511.5 (232.7, 1,155.1)
		Obesity	847.6 (422.4, 1,936.7)	198.3 (54.6, 3,310.1)	594.8 (321.8, 1,520.0)	841.9 (416.6, 1,902.2)	890.7 (479.9, 2,088.9)

Note: Costs were converted using 2023 conversion factors: exchange rate = 34.8022 THB per USD and purchasing power parity (PPP) conversion factor = 10.6468 THB per international dollar (Int$).
